# Identification of asthma phenotypes using blood cell count

**DOI:** 10.1016/j.ebiom.2022.103907

**Published:** 2022-02-25

**Authors:** Andriana I Papaioannou, Stelios Loukides, Petros Bakakos

**Affiliations:** a 2nd Respiratory Medicine Department Attikon’ University Hospital, National and Kapodistrian University of Athens, Medical School, Rimini 1, Chaidari, Athens 12462, Greece; b 1st Department of Pulmonary Medicine and Intensive Care Unit, National and Kapodistrian University of Athens, Medical School, Athens 115 27, Greece

Asthma is a common disease characterized by chronic airway inflammation.^1^ Although clinical manifestations among asthmatic patients are quite similar, asthma is a heterogeneous and complex disease in which several phenotypes have been described, mainly related to the age of onset, type and severity of symptoms, airway inflammation and treatment response.^2^ Since the identification of asthmatic phenotypes is of great importance for providing the correct and most effective therapeutic intervention, the development of easy, non-invasive, and affordable biomarkers is still an unmet need. In the latest years, the use of several biomarkers has been proposed^3^ while the identification of the type and number of inflammatory cells has been recognized as one of the most valuable approaches.

Since airway inflammation is a cardinal feature of asthma, the main inflammatory phenotypes have been described using the inflammatory cell profile in induced sputum samples. However, sputum induction and analysis are not easy for the patient, require expensive special laboratory equipment, qualified and trained staff and is not widely available. On the contrary, complete blood count is an inexpensive and precise test, routinely performed in everyday clinical practice, has a great reproducibility and is widely available. Furthermore, complete blood count may allow an estimation of the type of airway inflammation, since circulating eosinophil number has been shown to correlate to airway eosinophils^4^ (although we have to admit that this correlation is not strong) and has been reported that in the lung, approximately 100 tissue-residing eosinophils account for every blood eosinophil.^5^

In *eBioMedicine,* the study of Tsiavia T et al, reports the identification of asthma inflammatory phenotypes based on complete blood count using a French population based cohort.^6^ The authors have used the data of approximately 15.000 patients with current asthma from a large French population-based cohort and have identified four phenotypes based on the blood cell count. Importantly, among these phenotypes the most common was the low blood eosinophils/low blood neutrophils phenotype, accounting for 57% of patients and followed by the high blood eosinophil/low blood neutrophils, accounting for 33% of patients.

The absence of a definite increase of any type of inflammatory cells in the airways of asthmatic patients, also called as paucigranulocytic phenotype, has been recognized as one of the most common phenotypes in optimally treated asthmatic patients with an incidence which varies between 31 and 47% in different studies.^7^ In the great majority of these patients, asthma is characterized as “benign”, with a good response to therapy with corticosteroids probably related to the disappearance of eosinophilic inflammation. Similarly, in the study of Tsiavia et al, the low blood eosinophil/low blood neutrophil phenotype was associated with fewer day and night symptoms and fewer asthma exacerbations.^6^ However, it is known that approximately 10-15% of patients with paucigranulocytic asthma, representing 5–10% of the treated asthmatics, have a poor asthma control and are still at increased risk for asthma exacerbations.^7^ In these patients, it has been proposed that airway inflammation is probably related with different types of cells, such as macrophages or mast cells^8^ a fact that could also explain the reason that, regardless the absence of granulocytes, some of these patients show minimal or no response to therapy.^7^ Accordingly, in the study of Tsiavia et al a significant proportion of patients in the low blood eosinophil/low blood neutrophil phenotype still present a high asthma symptom score and report day and night symptoms and asthma attacks.^6^ Recognizing the possible different inflammatory pathways and the special clinical characteristics in this subgroup of patients of the paucigranulocytic phenotype, would probably result in increasing the potential for the development of novel therapeutic interventions resulting in the better management of these patients. However, we must point that the identification of paucigranulocytic asthma phenotype based on total blood count might not be always accurate as it has also been reported that inhaled corticosteroids might influence the number of blood eosinophils.^9^ The combined assessment of blood eosinophils with a more directed biomarker of airways eosinophilia FeNO could strengthen the inflammatory approach in a simpler way.

Importantly, approximately half of asthmatic patients included in the cohort of Tsiavia et al were overweight and approximately one third were uncontrolled.^6^ Obesity, increased age, and current smoking were associated to a high neutrophil count (regardless the number of blood eosinophils). Furthermore, the presence of high neutrophil count was also related to more severe symptoms, and presence of chronic bronchitis.^6^ Although the presence of concomitant COPD was not definitely excluded in this cohort, it seems that neutrophilic inflammation is probably related to a more systemic inflammatory process. However, we should always keep in mind that the elevation of neutrophil numbers might also be related to the use of inhaled corticosteroids.^10^

Overall, the study of Tsiavia et al shows that each inflammatory phenotype in asthma is associated with distinct clinical expression of the disease.^6^ The use of a test available in everyday clinical practice, such as the blood cell count, for recognizing the type of asthmatic inflammation for improving disease management and achieving total asthma control - despite how simplistic it may seem and taking under consideration the possible factors that affect the total blood cell count - is of great importance. Although clinical presentation in patients with asthma is quite similar, it seems that several underlying mechanisms might exist, resulting in differences in airway inflammation and response to treatment. The identification of easily accessible and affordable biomarkers is essential for the recognition of these different asthma phenotypes which would allow a better disease management and control.

## Declaration of interests

AIP has received honoraria for presentations and consulting fees from AstraZeneca, GlaxoSmithKlein, Novartis, Boehringer Ingelheim, Chiesi, and ELPEN. SL has received honoraria for presentations and consultancy fees from AstraZeneca, Boehringer Ingelheim, Chiesi, ELPEN, GlaxoSmithKlein, Menarini, Novartis and Sanofi, Specialty Therapeutics. PB has received honoraria for presentations and consultancy fees from AstraZeneca, Boehringer Ingelheim, Chiesi, ELPEN, GlaxoSmithKlein, Menarini, Novartis, Sanofi and Gilead.
